# Metastatic Melanoma Extending along the Pulmonary Vein into the Left Atrium: A Rare Route of Metastasis Characterized by Transesophageal Echocardiography

**DOI:** 10.1016/j.case.2022.04.007

**Published:** 2022-06-02

**Authors:** River Jiang, Dominique Kushneriuk, Michael H. Chiu

**Affiliations:** a Division of Cardiology, Gordon and Leslie Diamond Health Care Centre, University of British Columbia, Vancouver, British Columbia, Canada; b Department of Cardiology and Critical Care, Cummings School of Medicine, University of Calgary, Calgary, Alberta, Canada

**Keywords:** Cardiac metastases, Melanoma, Pulmonary vein infiltration, Left atrial mass

## Abstract

• Melanoma commonly metastasizes to the heart through hematogenous spread.
• These metastases can resemble benign cardiac tumors.
• Isolated left-sided cardiac involvement is rare with metastatic melanoma.
• Metastasis can atypically take a course through the pulmonary vein.
• Multimodality imaging is useful for characterization of these intracardiac masses.

## Graphical abstract

**Figure:**
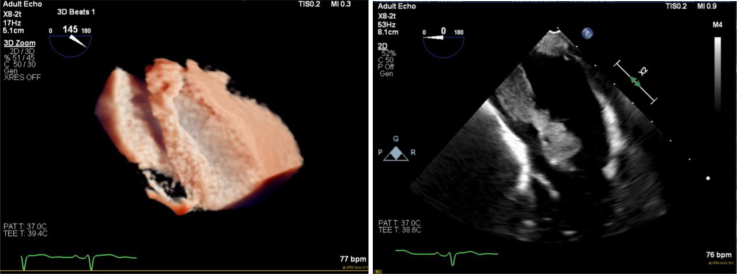

## Introduction

We report a case of a 48-year-old man who presented with an acute vestibular syndrome and was diagnosed with stage IV proto-oncogene B-Raf (BRAF) wild-type metastatic melanoma. Transthoracic echocardiogram (TTE) performed for stroke workup revealed a left atrial (LA) mass, which was subsequently characterized by transesophageal echocardiogram (TEE), computed tomography (CT), and positron emission tomography (PET). The metastatic lesion coursed from the pulmonary vein into the left atrium (LA), with disease also found in the lung, spleen, and adrenal gland. The patient was treated with anticoagulation, targeted immunotherapy, and external beam radiotherapy. Transthoracic echocardiogram follow-up 12.5 weeks after initiation of therapy showed resolution of the left atrial (LA) mass.

## Case Presentation

A 48-year-old man presented with several months of intermittent dizziness, blurred vision, instability with ambulation, nausea, and vomiting. He presented to the emergency department after an intense episode of vertigo that resulted in blunt trauma to his right shoulder and a severe left-sided headache. He did not recall experiencing any focal weaknesses but did describe left arm paresthesia. He had a stroke workup including a TTE that demonstrated an LA mass, so he was referred to the cardiology service.

### Physical Examination

On examination, his pulse rate was 89 beats per minute, with a blood pressure of 124/83 mm Hg. Multiple firm nontender submandibular and cervical lymph nodes were palpable on bilateral sides of his neck. Neurologic examination revealed left-beating nystagmus and right inferior quadrant hemianopsia. Cardiovascular examination was unremarkable, with no tumor plop on auscultation.

### Past Medical History

His medical history was significant for melanoma on the back resected 25 years before (Clark level 4, sentinel lymph node biopsy of right axilla negative), basal cell carcinoma on the right temple resected 2 years prior, and osteoporosis. His medications are naproxen and cyclobenzaprine, taken as needed.

### Differential Diagnosis

The differential diagnosis of an LA mass includes thrombus, tumor, or infection. Benign tumors, such as myxoma, which most commonly present in the LA as a pedunculated mass attached to fossa ovalis of the interatrial septum, can present with stroke symptoms, as was the case with this patient. Malignant tumor was high on the differential given the history of melanoma and the necrotic lymph nodes that were identified on the initial head CT imaging. Infection was low on the differential given the lack of infectious symptoms, lack of fever, and normal white blood cell count.

### Investigations

The patient’s laboratory evaluation revealed a white blood cell count of 6.9 (4-11 × 10^9^/L), hemoglobin, 135 (135-170 g/L), and normal renal function and electrolytes. His high-sensitivity troponin-I was 29 ng/L (<76 ng/L); D-dimer, 20,722 (<500 μg/L); C-reactive protein, 3.1 (<3.1 mg/L); and lactate dehydrogenase, 205 (90-240 U/L), normal values in parentheses. Two blood cultures demonstrated no growth after 5 days of incubation. Twenty-four-hour Holter monitor showed normal sinus rhythm, <1% premature atrial contractions, and no atrial fibrillation/flutter.

He was seen by the neurology service, and CT angiography of his head and neck was performed, which demonstrated infarcts of various ages involving the left cerebellum and left medial occipital lobe, as well as a 7.0 mm intra-axial high-attenuation lesion in the right frontal lobe with surrounding vasogenic edema, suspicious for hemorrhagic metastasis. In addition, there was extensive necrotic lymphadenopathy of the neck, with the largest measuring 13.0 mm.

Magnetic resonance imaging (MRI) of the head and spine confirmed the recent left posterior cerebral artery and left posterior inferior cerebellar artery territory infarcts. A right parietal enhancing lesion was seen, most consistent in appearance with a metastasis. In addition, there were additional numerous micrometastases versus tiny infarcts involving both cerebral hemispheres and a ventral C6-7 metastasis.

**Figure 1:**
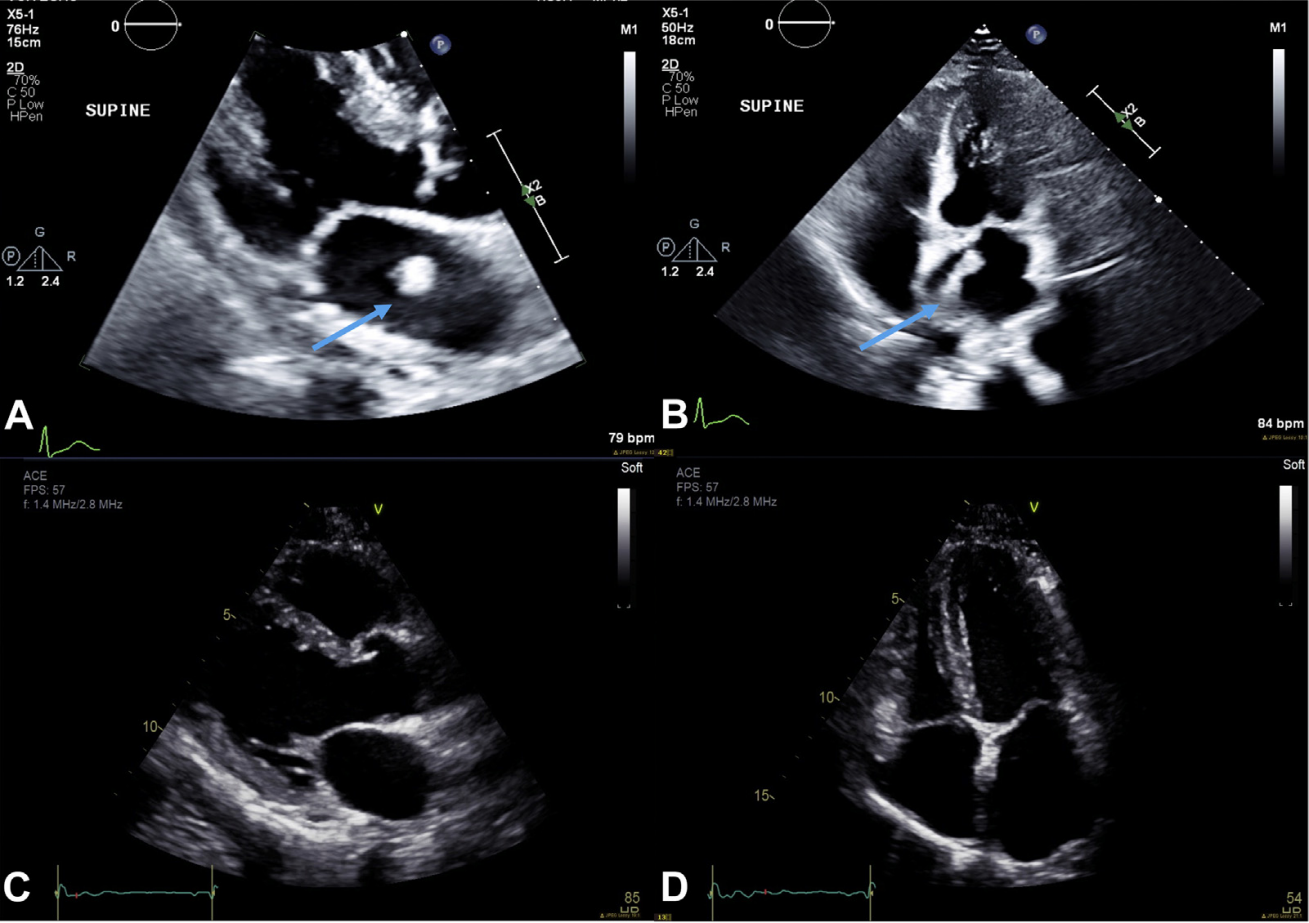
Transthoracic echocardiogram showing the parasternal long-axis *(left)* and 4-chamber views *(right)*. **(A-B)** Initial TTE with pedunculated stalk-like mass *(blue arrows)* with maximal orthogonal dimensions of 15 × 35 mm in the apical 4-chamber view and 15 × 12 mm in the 2-chamber views. **(C-D)** Transthoracic echocardiogram 12.5 weeks postdiagnosis and 10.5 weeks of therapy with no LA mass identified.

Transthoracic echocardiogram was performed as a part of his stroke workup. This revealed a homogenous, pedunculated mobile mass thought to be attached to the interatrial septum. The LA was mildly dilated by volume index (41 mL/m^2^). Otherwise, there was normal left ventricular size and systolic function, with visual left ventricular ejection fraction estimated at 60%; normal diastolic function and filling pressure with average E/e’ of 5.1, lateral e’ of 12.7 cm/sec, septal e’ of 9.2 cm/sec, trivial tricuspid regurgitation, and LA volume index of 41 mL/m^2^; no significant gradient across the mitral valve; and normal right ventricular size and systolic function (Figure 1A and B, Videos 1 and 2).

**Figure 2:**
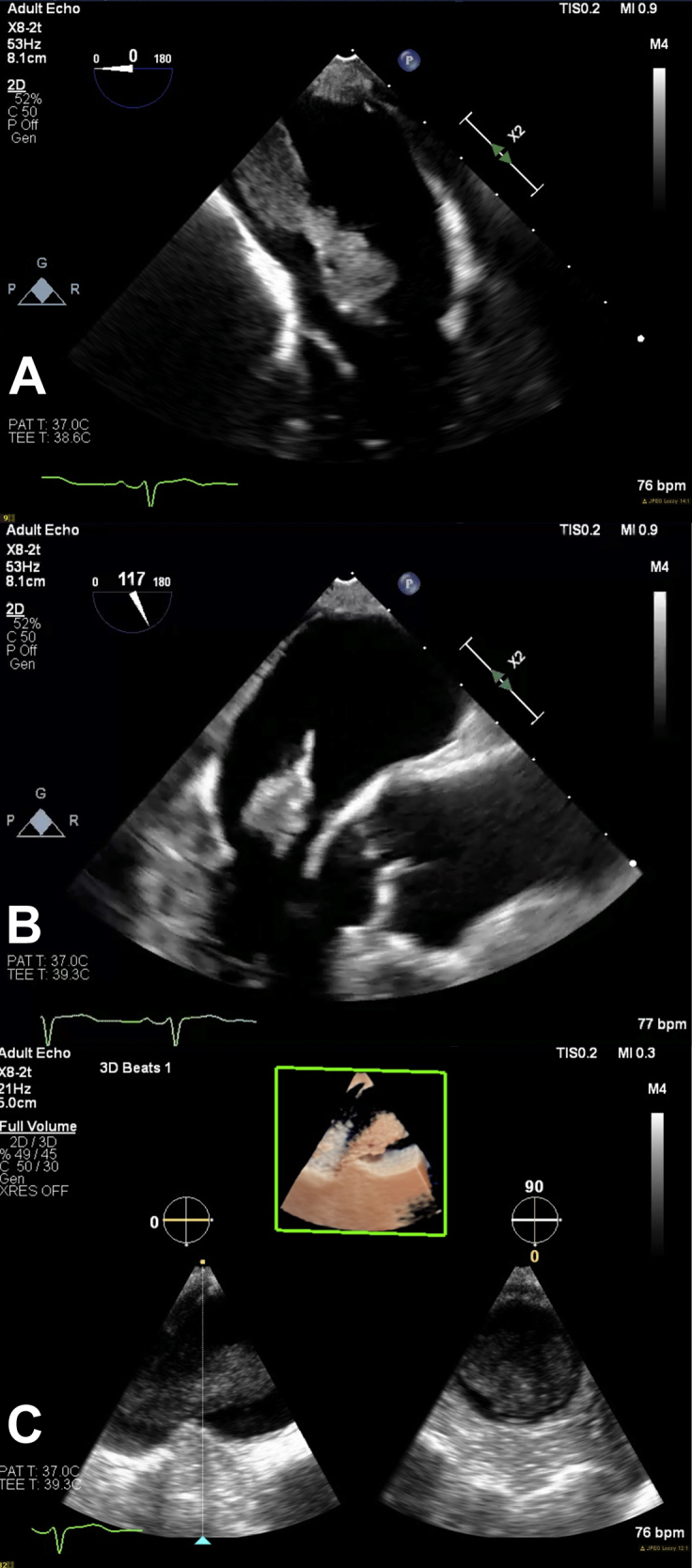
Transesophageal echocardiogram demonstrating a LA mass originating from the right lower pulmonary vein. The mass has a fixed component (39.0 × 13.3 mm) and a friable mobile segment (12.4 × 8.5 mm). **(A)** Four-chamber 0° zoom view of the LA mass. **(B)** Three-chamber zoom view at 117°. **(C)** X-plane of the mass originating from the right lower pulmonary vein.

**Figure 3:**
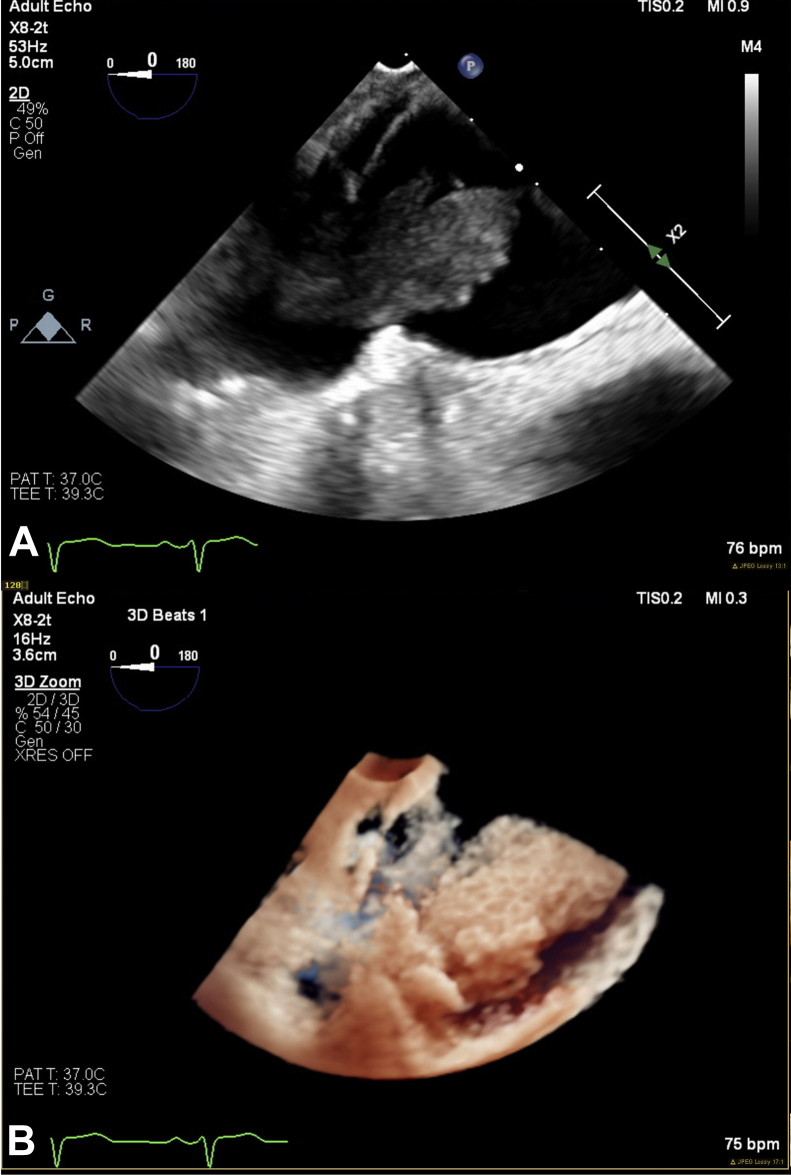
Transesophageal echocardiogram demonstrating an LA mass originating from the right lower pulmonary vein. The mass has a fixed component (39.0 × 13.3 mm) and a friable mobile segment (12.4 × 8.5 mm). Two-dimensional and associated three-dimensional TrueVue images. **(A)** Two-dimensional 0° view of the LA mass originating from the right lower pulmonary vein. **(B)** Three-dimensional TrueVue view of the LA mass from image **A**.

Transesophageal echocardiogram confirmed the large LA mass and demonstrated that it was originating from the right inferior pulmonary vein. The mass had a fixed component and a friable mobile segment measuring 12.4 × 8.5 mm. The fixed component appeared as a stalk and measured at least 39.0 × 13.3 mm. The mobile segment touched the mitral valve without causing obstruction (Figures 2 and 3, Videos 3-7). There was no LA appendage thrombus.

**Figure 4:**
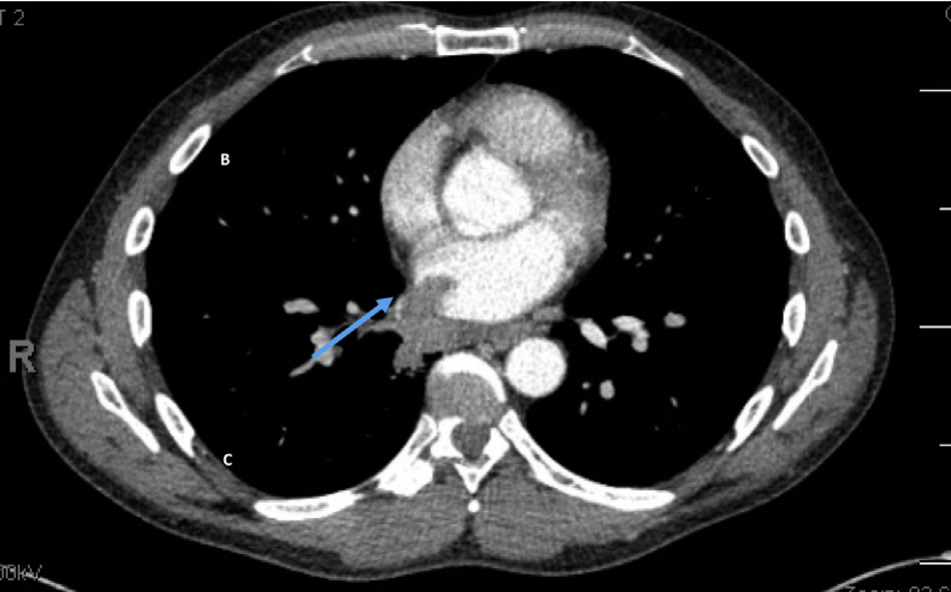
Contrast-enhanced CT scan demonstrating a solid tissue mass originating from the superior segment of the right lower lobe measuring 20 × 16 mm that invades the right lower pulmonary vein extending into the LA *(blue arrow)*.

Computed tomography chest, abdomen, and pelvis showed a solid soft tissue nodule in the superior segment of the right lower lobe measuring 20.0 × 16.0 mm. This was continuous with a mass that was invading the right inferior pulmonary vein and extending into the LA (Figure 4). Multiple enlarged hilar, interlobar, supraclavicular, axillary, and left retropectoralis lymph nodes were also noted.

**Figure 5:**
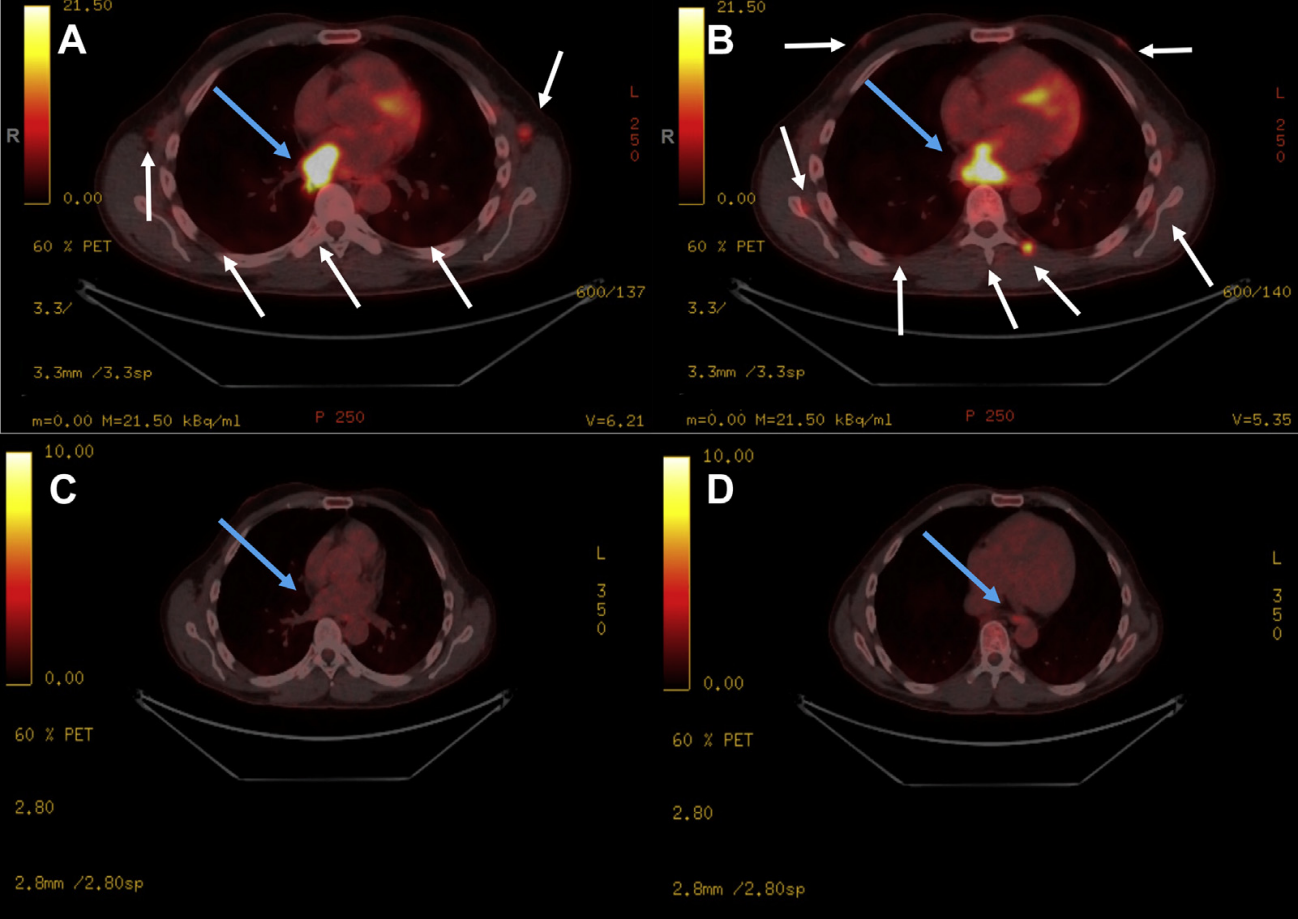
Positron emission tomography at the midthoracic cavity. **(A, B)** Initial PET demonstrating extensive nodal metastases above and below the diaphragm including a nodal metastasis in the distal paraesophageal region invading the right inferior pulmonary vein and extending into the LA *(blue arrows)*. There was small metastatic deposit at the left ventral C6/C7 spinal cord. There were solitary lung metastasis in the right lower lobe, innumerable subcutaneous and intramuscular deposits *(white arrows)*, and splenic and right adrenal metastases as well **(C, D)**. Repeat PET scan at 14 weeks showed further improvement of metabolic response with substantial improved bilateral posterior subpleural pulmonary opacities and decreased standardized uptake values max of the LA region and right inferior pulmonary vein of 2.5 and 6.5, decreasing from 8.1 and 9.7, respectively. *Blue arrows* are pointing to the nodal metastases invading the right pulmonary vein.

Positron emission tomography CT was performed; after a 6-hour fast, 281.3 MBq of ^18^F-fluorodeoxyglucose (FDG) was administered intravenously. Approximately 1 hour later, noncontrast CT and coregistered emission PET images were acquired from the vertex to the toes. This showed extensive nodal metastases above and below the diaphragm including a 34.0 × 31.0 mm FDG-avid nodal metastasis in the distal paraesophageal region invading the right inferior pulmonary vein and extending into the LA. There was no pericardial effusion or any other cardiac masses. There was a small metastatic deposit at the left ventral C6/C7 spinal cord. In addition, there were solitary lung metastasis in the right lower lobe, innumerable subcutaneous and intramuscular deposits, and splenic and right adrenal metastases (Figure 5A and B).

**Figure 6:**
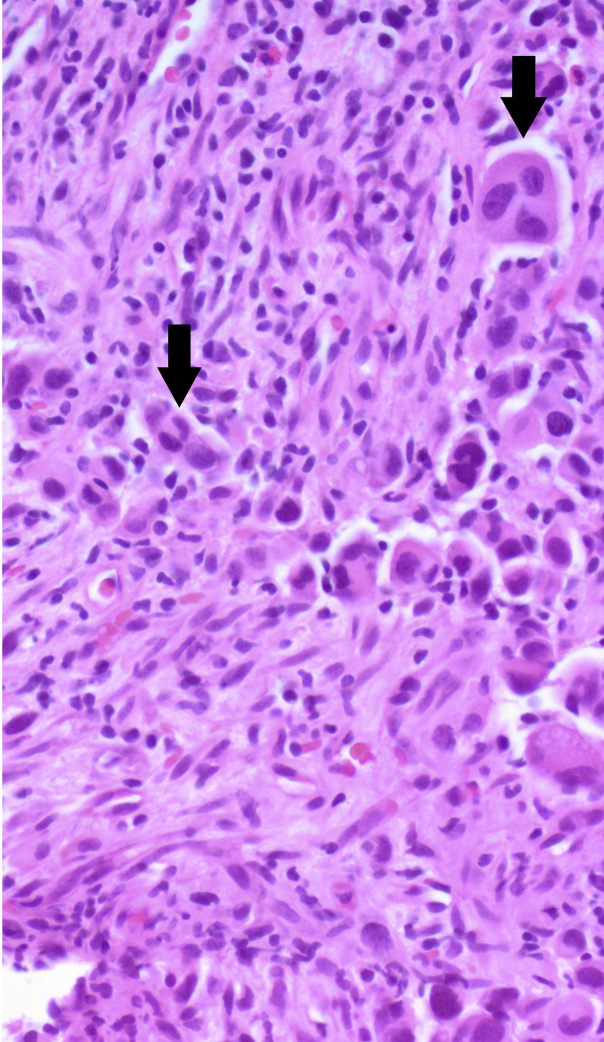
Ultrasound-guided core needle biopsy of the left submandibular lymph nodes. Histopathology showed large atypical nonpigmented epithelioid cells *(arrows)*.

An ultrasound-guided core needle biopsy of one of his left submandibular lymph nodes was performed. Histopathology showed large atypical nonpigmented epithelioid cells (Figure 6). Immunohistochemistry was consistent with metastatic melanoma (SOX10 positive, melan A negative, HMB45 negative, cytokeratin negative). Molecular analysis was BRAF V600E negative. Fungal culture, acid-fast bacillus (AFB) smear, and culture of the lymph node were negative.

## Management

After consultation with the hematology service and discussion with the patient, it was felt that his risk of continued embolization outweighed the risk of hemorrhagic transformation. He was initially started on therapeutic anticoagulation with dalteparin. He returned to the hospital 10 days later with headaches and emesis and was found to have 2 new parietal hemorrhagic metastases. The anticoagulation was held for 1 month and resumed at a lower prophylactic dose (5,000 units) by hematology once serial CT imaging showed no further intracranial hemorrhage. He was initiated on oral levetiracetam for presumed partial seizures due to brain metastases and hemorrhagic transformation. He completed external beam radiotherapy to the brain and C6-7 metastases (20 Gray × 5 fractions). Whole brain radiation was chosen over stereotactic or targeted radiotherapy due to the hemorrhagic nature and rapid changes in his brain metastases. He also received the first 2 cycles of ipilimumab and nivolumab, the first-line treatment for BRAF wild-type aggressive metastatic melanoma. This combination of immunotherapy has been shown to improve progression-free and overall survival compared to monotherapy.

## Follow-up

Medical treatment was initially well tolerated aside from the development of grade III hepatitis 7 weeks postimmunotherapy. This was managed with holding of his immunotherapy and a tapering course of glucocorticoids with resolution of his liver enzymes at 13 weeks. His only ongoing symptoms are intermittent low-intensity headaches and general fatigue.

A repeat PET/CT scan at 6 weeks showed substantial response to therapy with the right paraesophageal/right pulmonary vein/LA mass appearing smaller and less intense, and a PET/CT at 14 weeks demonstrated further improvement (Figure 5C and D). Similarly, repeat TTE performed at 12.5 weeks (10.5 weeks post-therapy) did not identify any LA mass (Figure 1C and D, Videos 8 and 9).

It was felt that the reduction in cardiac mass size was likely partially due to anticoagulation and partially due to immunotherapy. The current management plan is for follow-up PET/CT imaging and consideration of a resumption of immunotherapy if there is progression of disease.

## Discussion

We present a rare case of melanoma metastasizing to the LA through direct extension from a lung mass invading into and through the pulmonary vein, characterized by a multimodal imaging approach using TEE, PET, and CT.

The majority of cardiac masses are benign, and most represent myxomas.^1^ Other benign masses include septal lipoma. Cardiac masses due to malignancies are rare, with an estimated frequency of primary tumors <1/2,000.^2^ Primary tumors include undifferentiated sarcoma, osteosarcoma, and leiomyosarcoma. Metastatic tumors are 30 to 40 times more common, with autopsy series showing that the primary tumor most commonly originates from the lung, lymphoma, breast, leukemia, stomach, melanoma, liver, and colon.^3^ Tumors can spread to the heart by direct invasion (usually from the mediastinum), hematogenous spread, lymphatic spread, extension from the inferior vena cava, and, rarely, spread through the pulmonary veins.^1^

Melanoma has the greatest propensity for cardiac involvement out of all malignant tumors, with 50% of patients with melanoma having evidence of cardiac or pericardial metastasis based on autopsies^1^ and melanoma accounting for 34% of metastatic cardiac masses.^4^ Despite the high rate, cardiac involvement is rarely diagnosed antemortem due to lack of symptoms.

Cardiac metastatic melanomas usually arise from hematogenous spread,^4^ although they can also arise from transvenous spread through the inferior vena cava. Hematogenous metastases to the heart are usually associated with hematogenous metastases in other organs.^5^ All cardiac structures can be invaded; however, valve and endocardial involvement is uncommon. The most common sites are the epicardium, right atrium, and left ventricle.^6^ Direct invasion of a tumor to the heart typically occurs with bronchial, breast, and esophageal malignancies due to their close proximity to the heart. Direct transvenous extension can occur with primary lung carcinoma, thymic tumors, renal cell carcinoma, hepatocellular carcinoma, neuroblastoma, Wilms’ tumor, leiomyosarcoma, and adrenal adenocarcinoma. Malignant disease can resemble a benign cardiac tumor, especially in the context of an isolated atrial mass.^7^ A recent case report described a right atrial mass resembling a myxoma on TTE that was subsequently diagnosed as metastatic melanoma with advanced imaging and subsequently biopsy.^7^ This highlights the importance of a high clinical suspicion of malignant disease and the role of multimodality imaging.

Cardiac metastases from melanomas are frequently asymptomatic. When they cause symptoms, they are often nonspecific and include fatigue, dyspnea, weakness, palpitations, and chest pain. This can be associated with specific complications including pericardial effusion, obstructed right ventricular inflow or outflow, arrhythmias, congestive heart failure, stroke or transient ischemic attack, and, rarely, death.^7^ In a series of 70 autopsy cases, 42 had peritoneal, pleural, or pericardial effusion, 4 had a pericardial rub, and 57 had electrocardiographic abnormalities such as conduction disease, supraventricular tachycardia, or repolarization changes.^8^

Cardiac thrombi associated with metastatic melanoma have also been reported, but the prevalence is unknown.^9^ There is currently no guideline about prophylactic anticoagulation or antiplatelet therapy in the setting of cardiac thrombi with metastatic melanoma. A shared decision should be made with the patient considering the benefits and adverse events of potential treatment. Some factors on echocardiography may be suggestive of higher embolic potential, such as less echo-bright regions (that are softer and less well formed), mobile masses, or protruding or pedunculated thrombi.^10^

New cardiac symptoms in patients with malignancies or new embolic stroke symptoms should prompt further cardiac workup. Transthoracic echocardiogram is the initial modality of choice because of its high availability, cost-effectiveness, and lack of radiation exposure.^11^ Ultrasound-enhancing agents can help to define intracardiac masses from normal variants of cardiac anatomy. Differential opacification of the mass provides clues between vascularized and nonvascularized components.^12^ Computed tomography is useful for evaluating intraluminal filling defects and can provide a gross screen for myocardial/pericardial involvement. It also provides staging information as a part of a malignancy workup. In this case, both PET and CT imaging were useful for demonstrating the contiguous extension of the mass through the pulmonary vein to a likely pulmonary metastasis. Other imaging modalities for metastatic melanomas include MRI and fluorine-18-fluorodeoxyglucose (FDG) PET (where metastases are FDG avid).^13^

On cardiovascular MRI, metastases are typically T1 hypointense and heterogeneously T2 hyperintense; however, melanoma is an exception and is typically T1 hyperintense due to the presence of melanin.^14^ An MRI can also differentiate between thrombus and metastasis: whereas thrombus is avascular and does not show contrast enhancement, metastasis will show enhancement in both first-pass perfusion imaging and late gadolinium enhanced T1-weighted images.^14^

Prognosis of metastatic melanoma used to be extremely poor, with a 5-year overall survival of 10%. Evolution of therapeutic approaches including immunologic therapy has vastly improved survival. Immune check point inhibitors, cytotoxic T lymphocyte–associated protein 4 such as ipilimumab, and a programmed cell death protein 1 nivolumab are the most used immune check point inhibitors.

## Conclusion

Although cardiac involvement of metastatic melanoma is common, diagnosis antemortem usually does not occur because clinical signs are nonspecific and cardiac involvement can be masked by other visceral metastases. Cardiac involvement of melanoma is usually through hematogenous spread, associated with metastases elsewhere, and most commonly affects the epicardium/pericardium. Metastatic melanoma resembling an atrial myxoma, as well as manifesting as an isolated invasion into the pulmonary vein, is a rare presentation.^15^ Multimodality imaging including TEE, PET, and CT chest allowed for identification and localization of the cardiac mass.

## Supplementary Data

Video 1 Transthoracic echocardiogram showing the parasternal long-axis view with a pedunculated stalk-like mass measuring 14 × 11 mm.

Video 2 Transthoracic echocardiogram showing a pedunculated stalk-like mass measuring the largest at 15 × 35 mm in the apical 4-chamber view.

Video 3 Transesophageal echocardiogram 4-chamber 0° zoom view demonstrating an LA mass originating from the right lower pulmonary vein. The mass has a fixed component (39.0 × 13.3 mm) and a friable mobile segment (12.4 × 8.5 mm).

Video 4 Transesophageal echocardiogram 3-chamber 117° zoom view demonstrating an LA mass originating from the right lower pulmonary vein. The mass has a fixed component (39.0 × 13.3 mm) and a friable mobile segment (12.4 × 8.5 mm).

Video 5 Transesophageal echocardiogram X-plane of the mass originating from the right lower pulmonary vein.

Video 6 Transesophageal echocardiogram two-dimensional 0° view of the LA mass originating from the right lower pulmonary vein.

Video 7 Transesophageal echocardiogram three-dimensional 0° view of the LA mass originating from the right lower pulmonary vein.

Video 8 Transthoracic echocardiogram 12.5 weeks postdiagnosis and after 10.5 weeks of therapy with no LA mass identified in the parasternal long-axis view.

Video 9 Transthoracic echocardiogram 12.5 weeks postdiagnosis and after 10.5 weeks of therapy with no LA mass identified in the 4-chamber view.
